# Knowledge, Attitude, and Practice of Pregnant Women in Jazan, Saudi Arabia Concerning Pelvic Floor Muscle Exercises

**DOI:** 10.7759/cureus.28819

**Published:** 2022-09-06

**Authors:** Sarra L Derrar, Fatimah H Dallak, Azhar Alfaifi, Rawan M Alessa, Khawlah A Abbas, Atyaf J Zurayyir, Ahmed A Altraifi, Ibrahim Gosadi

**Affiliations:** 1 Department of Obstetrics and Gynecology, Faculty of Medicine, Jazan University, Jazan, SAU; 2 Department of Family and Community Medicine, Faculty of Medicine, Jazan University, Jazan, SAU

**Keywords:** knowledge assessment, saudi women, jazan region, pelvic floor muscle training, pelvic floor dysfunction

## Abstract

Introduction

Weaknesses of the pelvic floor muscles in females can lead to pelvic floor dysfunction thus increasing the risk of urinary and fecal incontinence. Furthermore, its weakness can lead to reproductive organ prolapse and sexual dysfunction, and influence sexual arousal and orgasm. There is limited evidence concerning the awareness of Saudi women regarding the importance of physical activity in the prevention and treatment of pelvic floor dysfunction. The current investigation aims to assess the knowledge, attitude, and practice of pelvic floor muscle exercises in the women of Jazan, Saudi Arabia.

Materials and methods

This investigation was a cross-sectional study targeting pregnant women in the Jazan region. Data was collected via an Arabic self-administered questionnaire. The questionnaire was composed of four main components measuring demographic data, presence of pelvic floor dysfunction symptoms, knowledge, attitude, and practice of the participants concerning pelvic floor muscle training. Chi-squared test was used to test the association between measured demographic factors and level of knowledge.

Results

A total of 183 pregnant women were recruited. The mean age of the participants was 27.4 years (standard deviation (SD): 5.3). The median number of pregnancies was 2 (interquartile range (IQR): 1-3), and the mean duration of current pregnancy was 20.9 (SD:11.1). The majority of pregnant women complained of lower abdomen pain with variable degrees. Nearly half of the sample complained of having either urinary or fecal incontinence with variable degrees of severity. The mean score of knowledge was 5 out of 12 and the scores varied between 1 and 11. Only 71 women (38.8%) were confident that pregnant women can exercise pelvic floor muscles. Furthermore, knowledge of the recruited mothers concerning the nature of the pelvic floor muscle exercise was relatively low in comparison to other items. Nearly one-third of the sample either believed that the exercise had no effect or had a worsening effect. Half the sample reported not performing any pelvic floor exercises. Only the practice of the exercise was statistically associated with the level of knowledge, indicating a higher proportion of women with a higher level of knowledge among those who regularly or occasionally perform the exercise. This may suggest that women with a higher level of knowledge are more motivated to exercise (<0.001).

Conclusion

Several deficiencies in knowledge about pelvic floor muscle exercise were detected among the recruited sample. Though the majority of the sample had an attitude favoring pelvic floor muscle exercise, nearly one-third did not believe pelvic floor muscle exercise can be beneficial. Finally, less than 10% of the sample indicated regular practice of pelvic floor muscle exercise. These findings indicate a need to increase the awareness and adherence of women in Jazan.

## Introduction

The pelvic floor is composed of several muscles and ligaments facilitating attachment to the bones of the pelvis. This combination creates a dome-shaped structure that provides support for several pelvic organs. These organs include the bladder and urethra, the vagina and uterus, and the rectum and anus [1]. Weaknesses of the pelvic floor muscles in females can lead to pelvic floor dysfunction thereby increasing the risk of urinary and fecal incontinence. Furthermore, as pelvic floor muscles provide physical support to the reproduction organs, its weakness can lead to reproductive organs prolapse [2], sexual dysfunction, and influence sexual arousal and orgasm [3].

The clinical definition of female pelvic floor dysfunction has been reported to vary. According to the International Urogynecological Association and the International Continence Society joint report on the terminology for female pelvic floor dysfunction, more than 250 separate definitions linked to pelvic floor muscle dysfunction were identified. However, the diagnoses of these conditions were related to main symptoms, including, urinary incontinence symptoms, bladder storage symptoms, sensory symptoms, voiding, and postmicturation symptoms, pelvic organ prolapse symptoms, sexual dysfunction symptoms, and anorectal dysfunction symptoms [4]. Due to variations in the clinical definition of pelvic floor dysfunction, different estimates of the prevalence of pelvic floor dysfunction have been provided [5]. For example, a US study that involved 1961 nonpregnant women indicated that 23.7% of them suffered a minimum of one pelvic floor disorder, of which, urinary incontinence was the most frequently reported [6]. Another Australian study that recruited 1517 women with a mean age of 71.5 years provided a higher prevalence of pelvic floor disorders where 47.2% of the recruited women has one or more pelvic floor conditions [7]. The risk of pelvic floor dysfunction has been reported to be linked to childbirth and aging [8].

Studies that assessed the prevalence of pelvic floor dysfunction in Saudi Arabia provided estimates varying between 17% and 60.2% [9-11]. These estimates indicate that pelvic floor dysfunction can be regarded as a common condition among Saudi women. The increase in the incidence of pelvic floor dysfunction among Saudi women has been linked to aging, greater parity, and assisted birth [10].

The high prevalence of pelvic floor dysfunction among Saudi women suggests a need for holistic curative and preventive measures. Management and prevention of pelvic floor dysfunction can involve surgical and non-surgical options [1]. Lifestyle modification, such as adopting a healthier diet, losing weight, and increasing physical activity, has been suggested to prevent and aid in the treatment of pelvic floor dysfunction. One of the physical activities that have been reported to increase the strength of pelvic floor muscles is pelvic floor muscle exercises [12]. Several reports indicated the impact of pelvic floor muscle exercises in the prevention of urinary and fecal incontinence [13-15].

Several factors have been reported to be associated with adherence to pelvic floor exercises, especially among vulnerable groups. In an Ethiopian study that involved 252 pregnant women, it was concluded that although 71% of them were taught about pelvic floor exercise, only 38.7% reported actual practice where the low practice levels were associated with tiredness, being busy, or forgetting to exercise [16]. Although studies that assessed awareness and practice of Saudi women concerning pelvic floor exercises are limited, similar estimates of the level of awareness and low practice level were identified in the Saudi community. A study conducted by Alharbi et al. that involved a sample of 152 postpartum women from the AlMadinah region indicated that although 62.7% of their sample was classified to have satisfactory knowledge about pelvic floor exercise, only 38.5% were correctly practicing them [17]. In an investigation that targeted primary healthcare physicians in the eastern region of Saudi Arabia to assess their awareness about exercise during pregnancy, most of the recruited 223 physicians (86.5%) were unaware of antenatal exercise clinical guidelines [18].

The current literature suggests a high prevalence of pelvic floor dysfunction conditions among Saudi women. Furthermore, there is limited evidence concerning the awareness of Saudi women about the importance of physical activity in the prevention and treatment of pelvic floor dysfunction. The current investigation aims to assess the knowledge, attitude, and practice of women in the south of Saudi Arabia about pelvic floor muscle exercise. Furthermore, this investigation aims to identify factors associated with the level of knowledge about pelvic floor muscle exercises.

## Materials and methods

Study context

This investigation was a cross-sectional study conducted in the Jazan region in the south of Saudi Arabia. Data collection was conducted between June and August 2022. Data collection was performed in primary healthcare settings in the Jazan, Sabya, Samtah, and Baish governorates of the Jazan region. Ethical approval to conduct the study was secured from the Jazan Health Ethics Committee (number 2244, dated 14/04/2022). Recruitment of the participants took place after securing their informed consent. No identification data were collected from the participants and data was collected in accordance with the declaration of Helsinki guidelines.

Data collection tool

Data was collected via an Arabic self-administered questionnaire. The questionnaire was composed of four main components. The first section asked the participants about their demographic data, and history of comorbidities, such as the presence of pelvic floor dysfunction conditions including lower abdomen pain, prolapse, urine, or fecal incontinence. The second section measured the knowledge of the participants concerning pelvic floor muscle training. The third section measured the attitude of the participants concerning pelvic floor muscle exercises. Finally, the fourth section measured the frequency of performing pelvic floor muscle exercises and the source of information about the exercises. Items used to compose the questionnaire and validation of the questionnaire were described elsewhere [19]. The questionnaire was piloted on a sample of mothers from the targeted community to test the face validity of the questionnaire.

Data collection process

Women were included in this investigation if they were pregnant at the time of participation. Women who were not pregnant at the time of recruitment were excluded. Ten primary healthcare centers were randomly selected from four governorates in Jazan. Identification of the participants was performed during antenatal clinic visits of the targeted women. After approaching and securing informed consent, mothers who were interested in participating were handed the questionnaire to have it filled, thus, completing the recruitment process. Sampling of the mothers was convenient and the required sample to perform the study was calculated via the StatCalc function of EpiInfo software from the Centers for Disease Control and Prevention (CDC, Atlanta, USA). By utilizing a prevalence of 62.7% reported by Alharbi et al., classifying women as having a satisfactory level of knowledge about pelvic floor muscle exercises [17], 90% confidence level, and 5% margin of error, a sample of 253 pregnant women was estimated.

Data analysis

Data analysis was performed via the Statistical Package for Social Sciences software, version 21 (IBM Corp., Armonk, NY). Frequencies and proportions were used to summarize binary and categorical variables while means, medians, and standard deviations (SD) were used to summarize continuous variables according to the distribution of each continuous variable. Scoring of knowledge of the participants was conducted by giving each correct answer a score of one. Summing the scores was performed to calculate the median, minimum, and maximum values of knowledge scores due to the abnormal distribution of the knowledge scores. The average value was used to classify the sample into women with higher and lower levels of knowledge about pelvic floor muscle exercise. Chi-squared test was used to test the association between measured demographic factors and level of knowledge. Due to the small sample size, the following variables were grouped to avoid having empty cases in the given categories. These include grouping of age, pregnancy duration, and number of pregnancies according to the median values. Additionally, education level was grouped as having less than university education or university level; employment was grouped as employed or being a housewife. A p-value of 0.05 was presumed as a statistically significant value for the applied statistical tests.

## Results

The total number of identified women was 233, of whom, 50 women were excluded due to reasons which were either refusing to participate or not meeting the inclusion criteria of being pregnant at the time of recruitment. The total number of pregnant women who were recruited was 183. The mean age of the participants was 27.4 years (SD: 5.3). The median of the number of pregnancies was 2 (IQR: 1-3), and the mean of the duration of current pregnancy was 20.9 (SD:11.1). The majority of the participants had university level education (72.1%), and more than half were housewives (55.7%).

The majority of pregnant women complained of lower abdomen pain with variable degrees. Nearly half of the participants complained of having either urinary or fecal incontinence with variable degrees of severity. However, the majority (86.9%) did not report symptoms indicating reproductive organ prolapse (Table 1).

**Table 1:** Demographic characteristics and presence of pelvic floor dysfunction symptoms among 183 pregnant women from Jazan, Saudi Arabia

Variable	
Age: mean [SD]	27.4 [5.3]
Number of pregnancies: median [IQR]	2 [1-3]
Current pregnancy duration in weeks: mean [SD]	20.9 [11.1]
Education: Frequency [proportion]	
Primary	1 [0.5%]
Intermediate	8 [4.4%]
Secondary	42 [23%]
University	132 [72.1%]
Employment: Frequency [proportion]	
Governmental	36 [19.7%]
Private	24 [13.1%]
Housewife	102 [55.7%]
Business owner	21 [11.5%]
Do you have pain in the lower abdomen?: Frequency [proportion]	
No	51 [27.9%]
Yes, but without affecting everyday life	48 [26.2%]
Yes, slight pain	44 [24%]
Yes, moderate pain	35 [19.1%]
Yes, strong pain	5 [2.7%]
Do you have a lump emerging from your vagina? Frequency [proportion]	
No	159 [86.9%]
Yes, but without affecting everyday life	8 [4.4%]
Yes, it has a slight effect	9 [4.9%]
Yes, it has moderate effect	5 [2.7%]
Yes, it has strong effect	2 [1.1%]
Do you have urinary incontinence problems? Frequency [proportion]	
No	90 [49.2%]
Yes, but without affecting everyday life	29 [15.8%]
Yes, it has a slight effect	31 [16.9%]
Yes, it has moderate effect	22 [12%]
Yes, it has strong effect	11 [6%]
Do you have problems with defecation? Frequency [proportion]	
No	93 [50.8%]
Yes, but without affecting everyday life	32 [17.5%]
Yes, it has a slight effect	25 [13.7%]
Yes, it has moderate effect	21 [11.5%]
Yes, it has strong effect	12 [6.6%]

The mean score of knowledge was 5 out of 12 and the scores varied between 1 and 11 (Table 2). Only 71 women (38.8%) were confident that pregnant women can exercise pelvic floor muscles. Furthermore, knowledge of the recruited mothers concerning the nature of the pelvic floor muscle exercises were relatively low in comparison to other items. Finally, it can be noted that awareness of recruited mothers about the benefit of pelvic floor muscle exercises is higher in relation to the items measuring the nature of the exercise.

**Table 2:** Knowledge about pelvic floor muscle exercises among 183 pregnant women from Jazan, Saudi Arabia *Correct answers

Item	Frequency [proportion]
What area of muscles do you think exercising the pelvic floor muscles is a great practice?	
Muscles around the genitals and anus*	59 [32.2%]
Abdominal muscles	12 [6.6%]
Front leg muscles	4 [2.2%]
Back muscles	4 [2.2%]
I don’t know	104 [56.8%]
When exercising the pelvic floor muscles, how should you breathe?	
Breathing normally while contracting the muscles*	18 [9.8%]
Hold your breath while contracting the muscles	10 [5.5%]
Inhale while contracting the muscles and exhale while loosing	37 [20.2%]
I don’t know	118 [64.5%]
While exercising the pelvic floor muscle, abdominal muscles should be contracted together.	
True	29 [15.8%]
False*	21 [11.5%]
I don’t know	133 [72.7%]
Proper posture while exercising pelvic floor muscle is:	
Sleeping position only	19 [10.4%]
Sitting position only	17 [9.3%]
Standing position only	7 [3.8%]
Any position*	31 [16.9%]
I don’t know	109 [59.6%]
The benefit of exercising the pelvic floor muscles is:	
Reduces incidence of urinary incontinence and facilitate birth*	84 [45.9%]
Reduces incidence of constipation	1 [0.5%]
I don’t know	98 [53.6%]
The disadvantage of exercising the pelvic floor muscles is	
Can cause sexual intercourse pain	8 [4.4%]
Can cause difficulty of defecation	2 [1.1%]
Can cause pelvic pain	13 [7.1%]
There are no disadvantages*	35 [19.1%]
I don’t know	125 [68.3%]
Can pregnant women exercise the pelvic floor muscles?	
Yes*	71 [38.8%]
No	6 [3.3%]
I don’t know	106 [57.9%]
Pelvic floor muscle exercise can cause preterm birth?	
True	45 [24.6%]
False*	138 [75.4%]
Pelvic floor muscle exercise can facilitate easier birth?	
True*	164 [89.6%]
False	19 [10.4%]
Pelvic floor muscle exercise can cause difficult birth?	
True	13 [7.1%]
False*	170 [92.9%]
Pelvic floor muscle exercise can cause baby death due to lack of oxygen?	
True	22 [12%]
False*	161 [88%]
Pelvic floor muscle exercise can reduce risk of vaginal tear?	
True*	116 [63.4%]
False	67 [36.6%]
Mean score [SD]: 5/12 [2.1]. Minimum- Maximum scores: 1-11	

Majority of the sample had a positive attitude toward the exercise (Table 3). However, nearly one-third of the sample either believed that the exercise had no effect or had a worsening effect. When the women were asked about what motivated them to initiate the exercise, the most frequent factor was influence of the media (21.3%), and their treating physicians (18.6%). Furthermore, when the mothers were asked about factors that kept them motivated to continue the exercise, the main reason was observing the benefits of the exercise (38.9%) followed by fear of performing surgery to treat conditions associated with pelvic floor dysfunction (23.3%) (Table 4). Finally, only the practice of the exercise was statistically associated with the level of knowledge, indicating a higher proportion of women with a higher level of knowledge among those who regularly or occasionally performed the exercise (Table 5). This may suggest that women with higher levels of knowledge are more motivated to exercise (P value <0.001). However, due to the retrospective nature of the investigation, it was not possible to confirm the temporality of this association.

**Table 3:** Attitude of 183 pregnant women from Jazan, Saudi Arabia concerning pelvic floor muscle exercises

Statement	Yes, large improvement	Yes, slight improvement	No effect	Yes, worsening effect
Do you think that pelvic floor muscle exercise can influence sexual emotions?	61 [33.3%]	60 [32.8%]	57 [31.1%]	5 [2.7%]
Do you think that pelvic floor muscle exercise can influence sexual satisfaction?	62 [33.9%]	57 [31.1%]	59 [32.2%]	5 [2.7%]
Do you think that pelvic floor muscle exercise can influence pelvic pain?	72 [39.3%]	65 [35.5%]	39 [21.3%]	7 [3.8%]
Do you think that pelvic floor muscle exercise can influence stress urinary incontinence?	63 [34.4%]	64 [35%]	50 [27.3%]	6 [3.3%]
Do you think that pelvic floor muscle exercise can influence urgency urinary incontinence?	56 [30.6%]	67 [36.6%]	56 [30.6%]	4 [2.2%]
Do you think that pelvic floor muscle exercise can influence urinary retention?	57 [31.1%]	62 [33.9%]	62 [33.9%]	2 [1.1%]
Do you think that pelvic floor muscle exercise can influence pelvic organ prolapse?	57 [31.1%]	61 [33.3%]	63 [34.4%]	2 [1.1%]
Do you think that pelvic floor muscle exercise can influence constipation?	43 [23.5%]	67 [36.6%]	64 [35%]	9 [4.9%]
Do you think that pelvic floor muscle exercise can influence fecal incontinence?	41 [22.4%]	67 [36.6%]	68 [37.2%]	7 [3.8%]
Do you think that pelvic floor muscle exercise can influence general health?	78 [42.6%]	67 [36.6%]	33 [18%]	5 [2.7%]

**Table 4:** Practice and motivators of pelvic floor muscle exercises among 183 pregnant women from Jazan, Saudi Arabia *Participants were able to choose more than one answer

Item	Frequency [proportion]
How often do you perform pelvic floor muscles exercise?	
Regularly	12 [6.6%]
Occasionally	78 [42.6%]
Do not perform any	93 [50.8%]
What motivates you to initiate pelvic floor muscle exercise*	
Husband	31 [16.9%]
Family members	22 [12.1%]
Friends	19 [10.4%]
Physicians	34 [18.6%]
Hospital educational materials	16 [8.7%]
Hospital staff	10 [5.5%]
Social media, websites, TV or radio	39 [21.3%]
What factors keeps you motivated to perform pelvic floor muscles exercise?	
Getting reminders from certain people	21 [11.7%]
Observing benefits of the exercise	70 [38.9%]
Prevention of disease recurrence	10 [5.6%]
Disease prevention	33 [18.3%]
Fear of performing the surgery	42 [23.3%]
Other	4 [2.2%]
Missing	3 [1.6%]

**Table 5:** Factors associated with knowledge level about pelvic floor muscle exercises among 183 pregnant women from Jazan, Saudi Arabia

Factor	Lower knowledge	Higher knowledge	P value
Age			1.000
27 years or less	58 [56.3%]	45 [56.3%]	
More than 27 years	45 [43.7%]	35 [43.8%]	
Total	103 [100%]	80 [100%]	
Education			1.000
Less than university education	29 [28.2%]	22 [27.5%]	
University education	74 [71.8%]	58 [72.5%]	
Total	103 [100%]	80 [100%]	
Employment			0.764
Housewife	56 [54.4%]	46 [57.5%]	
Employed	47 [45.6%]	34 [42.5%]	
Total	103 [100%]	80 [100%]	
Current pregnancy duration			0.883
21 weeks or less	54 [52.4%]	41 [51.2%]	
More than 21 weeks	49 [47.6%]	39 [48.8%]	
Total	103 [100%]	80 [100%]	
Number of pregnancies			0.763
Two or less	61 [59.2%]	45 [56.3%]	
More than two	42 [40.8%]	35 [43.8%]	
Total	103 [100%]	80 [100%]	
Pelvic floor muscles exercise			<0.001
Regularly	3 [2.9%]	9 [11.3%]	
Occasionally	27 [26.2%]	51 [63.7%]	
Never	73 [70.9%]	20 [25%]	
Total	103 [100%]	80 [100%]	

## Discussion

This was a cross-sectional investigation targeting pregnant women in the Jazan region of Saudi Arabia to measure their knowledge, attitude, and practice concerning pelvic floor muscle exercises. The majority of the measure had good knowledge and attitude concerning the benefits of pelvic floor exercise. However, nearly one-third of the sample did not believe that pelvic floor muscle exercises are beneficial and less than 10% of the sample reported performing the exercises on a regular basis. When the women were asked about what motivates them to exercise, more than 20% indicated that the media was the most influential. None of the demographic factors was associated with the level of knowledge about pelvic floor muscle exercises except the association between the knowledge and practice level.

The findings of the current investigation can be compared to similar national and international investigations. Nonetheless, studies that measured the level of awareness of women in Saudi Arabia concerning pelvic floor muscle exercises are limited. Al-Rowais et al. performed an online investigation targeting pregnant women in Saudi Arabia and was able to recruit 399 pregnant women to measure their knowledge, attitude, and practice toward pelvic floor exercises. While considering the detected methodological differences between the study by Al-Rowais et al. and the current investigation, Al-Rowais et al. concluded a low level of knowledge among their sample. For example, 33.1% of their sample believed that pelvic floor muscle exercises are harmful to their infants and thus discouraged them from performing the exercise [20]. Our study detected a lower level of knowledge concerning items measured in the nature of pelvic floor muscle exercises. However, barriers to the exercise were not measured in our investigation, yet nearly 40% of our sample reported being motivated to exercise because they did observe benefits from the exercise.

Alharbi et al. conducted a cross-sectional investigation recruiting 152 postnatal women from Almadinah Almunawarah to measure their knowledge, attitude, and practice of Kegel exercise. Although Alharbi et al reported that more than half of their sample had satisfactory knowledge about the exercise, and more than 70% had an attitude favoring the exercise, only 38.5% of their sample were adequately performing the exercise which is similar to our findings, especially with regard to the low practice level detected in our sample. Furthermore, nearly one-quarter of their sample had their information about the Kegel exercise from social media [17]. This notion is similar to the current investigation where 20% of our sample reported media as an influence in motivating them to perform the exercise.

Similar international investigations that targeted pregnant women were identified. Temtanakitpaisan et al. performed a cross-sectional investigation involving a sample of 110 pregnant women from Thailand to assess their knowledge, attitude, and practice concerning pelvic floor muscle exercises. Temtanakitpaisan et al. reported that despite having a high level of knowledge about the importance of the exercise, only 10.7% regularly performed the exercise [19]. This finding is similar to ours which can be justified by the presence of barriers affecting women’s willingness to exercise. This is supported by the findings of another similar study conducted in Nigeria with a sample of 252 pregnant women which indicated that despite the engagement of their sample in the education program about pelvic floor muscle exercises, only 38.3% practiced the exercise. Furthermore, it was concluded that forgetting about the exercise, tiredness, and lack of time were the main barriers to the exercises [16].

Our investigation did not detect associations between demographic factors and level of knowledge about pelvic floor muscle exercises which can be partially explained by the low level of knowledge concerning certain items affecting the overall knowledge score. Nonetheless, a study involving 169 Pakistani women indicated that educational status was positively correlated with the level of awareness about pelvic floor muscle exercises [21]. Furthermore, another Ethiopian study with a sample of 349 pregnant women assessed factors associated with antenatal exercises, including pelvic floor muscle exercises; they reported that education level, employment, and being advised on exercising during antenatal care were associated with the knowledge, attitude, and practice levels of antenatal exercise [22].

Strengths and limitations

Our investigation has multiple areas of strengths and limitations. The main strengths of the current study are related to the targeted sample of pregnant women who are likely to be affected by the ramifications of pelvic floor dysfunction at the time of recruitment and can be considered a target group for pelvic floor muscle exercises education. The limitations of the current investigation are related to the inherent nature of the data collection tool which relies on the recall of the participants. Furthermore, the inclusion criteria of limiting the sample to pregnant women, at the time of recruitment, affected reaching a larger sample size. Finally, utilization of the cross-sectional design is a limitation to the current investigation, influencing the temporal association of dependent and independent factors.

## Conclusions

Several deficiencies in knowledge about pelvic floor muscle exercises were detected among the study participants. Though the majority of the sample had an attitude favoring pelvic floor muscle exercises, nearly one-third of the sample did not believe that pelvic floor muscle exercises can be beneficial. Finally, less than 10% of the sample indicated regular practice of the pelvic floor muscles exercise. The finding indicates a need for efforts to increase the awareness and adherence of women in Jazan with regard to pelvic floor muscle exercises, especially when considering the reliance of women on media or family members as motivators to initiate the exercise, thereby suggesting a limited role of healthcare establishments. Nearly half of the sample reported suffering from pelvic floor dysfunction with variable degrees. Since our sample was limited to pregnant women and not representative of the general women of Jazan, there is a need for further research to identify the prevalence and factors associated with pelvic floor dysfunction in the region.
